# Detectability Counts when Assessing Populations for Biodiversity Targets

**DOI:** 10.1371/journal.pone.0024206

**Published:** 2011-09-27

**Authors:** Silviu O. Petrovan, Alastair I. Ward, Philip Wheeler

**Affiliations:** 1 Centre for Environmental and Marine Sciences, University of Hull, Scarborough Campus, Scarborough, United Kingdom; 2 The Food and Environment Research Agency, Sand Hutton, York, United Kingdom; Texas A&M University, United States of America

## Abstract

Efficient, practical and accurate estimates of population parameters are a necessary basis for effective conservation action to meet biodiversity targets. The brown hare is representative of many European farmland species: historically widespread and abundant but having undergone rapid declines as a result of agricultural intensification. As a priority species in the UK Biodiversity Action Plan, it has national targets for population increase that are part of wider national environmental indicators. Previous research has indicated that brown hare declines have been greatest in pastural landscapes and that gains might be made by focussing conservation effort there. We therefore used hares in pastural landscapes to examine how basic changes in survey methodology can affect the precision of population density estimates and related these to national targets for biodiversity conservation in the UK. Line transects for hares carried out at night resulted in higher numbers of detections, had better-fitting detection functions and provided more robust density estimates with lower effort than those during the day, due primarily to the increased probability of detection of hares at night and the nature of hare responses to the observer. Hare spring densities varied widely within a single region, with a pooled mean of 20.6 hares km^−2^, significantly higher than the reported national average of hares in pastures of 3.3 hares km^−2^. The high number of encounters allowed us to resolve hare densities at site, season and year scales. We demonstrate how survey conduct can impact on data quantity and quality with implications for setting and monitoring biodiversity targets. Our case study of the brown hare provides evidence that for wildlife species with low detectability, large scale volunteer-based monitoring programmes, either species specific or generalist, might be more successfully and efficiently carried out by a small number of trained personnel able to employ methods that maximise detectability.

## Introduction

Effective conservation action relies on setting of achievable targets, ideally based on sound science and reliable data. At its most fundamental level this depends on adequate, efficient assessments of the status of populations [1]. Such population assessments should be cost-effective and practicable, but in order to provide information that is usable by conservation managers and decision makers they should also estimate population parameters at a sufficient scale and with sufficient precision to permit detection of changes in these that are relevant to policy development [2]. National or continental-scale population surveys pose particular problems because of the spatial scale at which they must be carried out, requiring substantial data collection with sufficiently detailed spatial coverage. There are a number of well-established large-scale population monitoring programmes for, among others European butterflies [3], British breeding birds [4] and North American birds [5], but despite efforts to co-ordinate available data in the United Kingdom [6] as yet, long-term national surveys of mammals are few and unvalidated.

In many countries, including the UK, governments employ headline indicators to assess progress in sustainable development as a basis of informing and shaping policy (http://www.defra.gov.uk/sustainable/government/). One such indicator of ‘Biodiversity Conservation’ is based partly on an assessment of changes in population size and trends of priority species under the UK Biodiversity Action Plan (UK BAP). Adequate assessments of population changes in priority species are therefore a key component of national biodiversity policy and a wider governmental sustainable development agenda.

The brown hare *Lepus europaeus* is an iconic species of European farmland regarded as an indicator for the habitat quality of lowland agricultural landscapes [7], a popular game species and a “priority species of conservation concern” in the UK BAP [8]. Hare populations appear to have undergone a marked decline across Europe in the second half of the 20^th^ century [9], [10], [11] and in Britain this decline has been particularly marked in pastural landscapes [12]. Partial recovery of the brown hare population was a key aim of the UK BAP, with a target of doubling 1996 numbers by 2010 [8]. The 1996 assessment of the population size of hares was based on the ‘national hare survey’, a country-wide survey using volunteers to carry out line-transect distance sampling of resting hares during the daytime [13]. This survey was a landmark in wildlife monitoring in Britain as it was the first attempt to provide a country-wide population estimate of a terrestrial mammal based on a properly stratified sample of survey sites and designed as part of a programme to measure performance against a defined policy objective [8], [13]. The method for the survey, daytime line transect distance sampling, was selected on the basis of practicality, robustness, ability to incorporate wide spatial coverage and to allow participation by volunteers [13], [14]. Distance sampling has been widely used as a tool for monitoring a range of wildlife species in recent years and is considered to be more robust than some other methods because it does not assume that all animals present at the time of the survey will be observed. It has been shown to produce reliable abundance estimates in a variety of terrestrial mammalian species including brown hare [13], [14], [15], mountain hare *Lepus timidus* [16] and Irish hare *Lepus timidus hibernicus* [17], [18].

The national hare survey's results indicated that hares occurred at mean densities of 3.3 hares km^−2^ in pastures and 2.5 hares km^−2^ in marginal upland and identified significant opportunities for increasing the British hare population through improving the quality of hare habitat in such landscapes, rather than arable-dominated regions where the species can be a pest and densities were already comparatively high [13]. However, because relatively few hares (400) were seen in pastural and marginal upland areas, despite over 3,000 km of survey effort, the survey was unable to resolve hare densities at a ‘habitat within region’ scale. The methods employed in this first survey were therefore not able to deal with the key management questions that the results of the survey raised.

Brown hares are largely crepuscular and nocturnal, resting in a ‘form’ during the day [19]. Night time surveys were rejected as a method in the national survey for reasons of logistical feasibility and to avoid the possibility that active animals would move prior to observation or remain concealed in tall vegetation. However, attempting to count potentially concealed inactive animals is challenging as their detectability is likely to be significantly lower than active individuals. We considered *a priori* that a hypothesised increased detectability of hares at night would increase encounter rates and facilitate greater spatial and temporal resolution of population density estimates from distance sampling with consequences for the spatial and temporal scale of monitoring and, by implication, target-setting. This study therefore had three aims:

1. To carry out a quantitative assessment of differences in population estimation between daytime and night time line-transect distance sampling surveys of brown hares.
2. To carry out a regional scale survey of brown hares in a pasture-dominated region.
3. To attempt to resolve densities of hares at seasonal and sub-regional scales.

## Results

### Night time versus daytime encounter rates and densities

**Figure 1:**
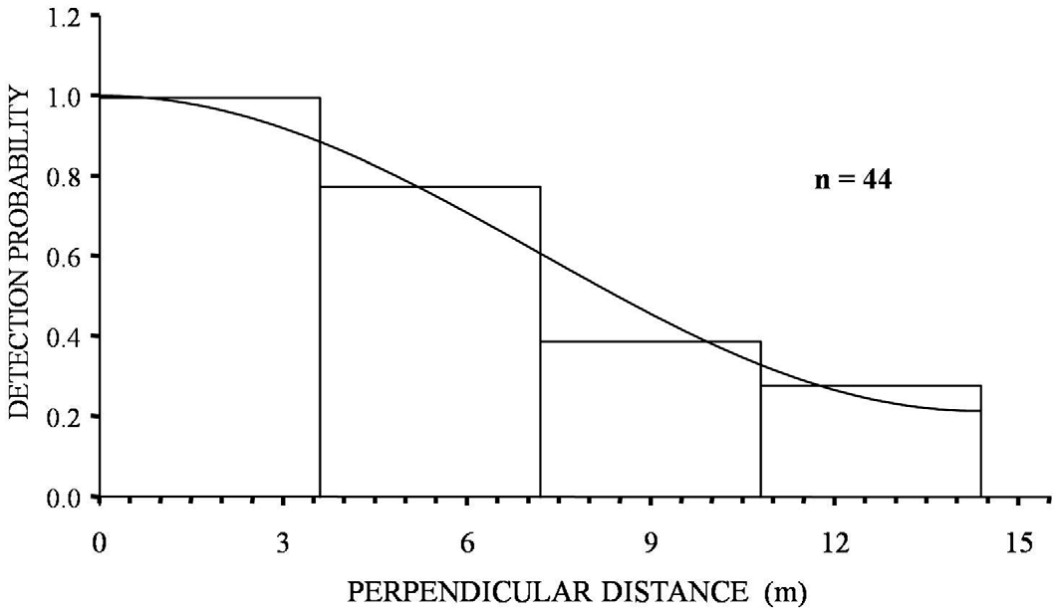
Histogram of total daytime hare observations in the two sites surveyed three times during winter 2008–2009. Fitted model is a Uniform key function with a cosine adjustment term, no intervals, no truncation.

**Figure 2:**
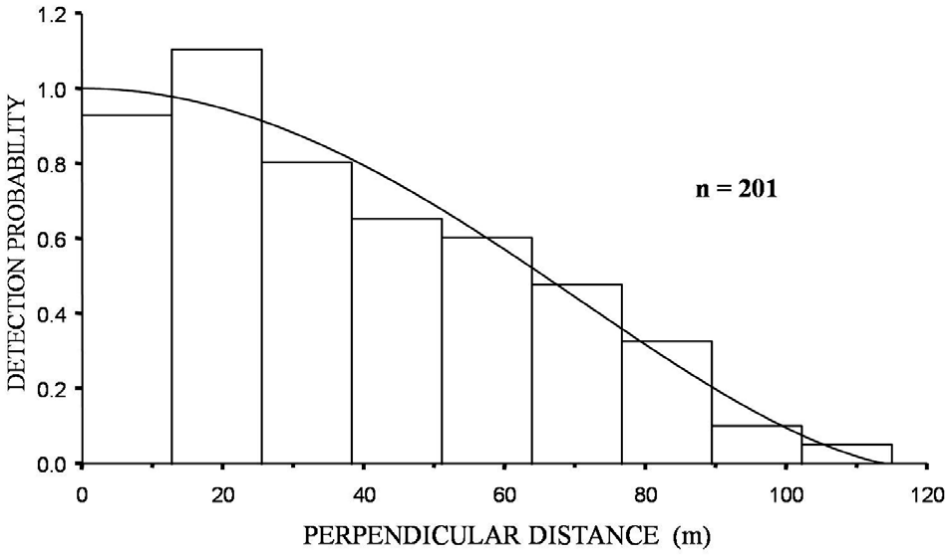
Histogram of total night time hare observations in the two sites surveyed twice during winter 2008–2009. Fitted model is a Uniform key function with a simple polynomial adjustment term, no intervals, 115 m right truncation.

Daytime encounter rates (0.7 hares km^−1^; 95% CI 0.5–0.9) were 7.4 times lower than night time encounter rates (5.2 hares km^−1^; 95% CI 4.2–6.5) on the same transects (Table 1). Effective strip width (ESW) was much lower in daytime than night time surveys (8.9 m and 64.4 m respectively; Figures 1 and 2). Mean pooled density estimates for daytime surveys for the two sites (41.2 hares km^−2^; 95% CI 29–59 km^−2^) were considerably higher than night time estimates (32.3 hares km^−2^; 95% CI 26–40 km^−2^) but the very large confidence intervals for daytime estimates, resulting from the low daytime encounter rate, meant that the results of a Z-test of differences between the daytime and night time estimates was not significant (Z = 1.01, P = 0.31).

**Table 1:** Estimates of hare density, effort and encounter rates in the two sites surveyed during winter 2008–2009 (calculated with Hazard-rate key function, cosine adjustment term, 16 m interval, 115 m truncation for night time surveys and Uniform key function with cosine adjustment term, no interval, no truncation for daytime surveys).

Site	Total effort (km)	Total transects	Hares seen	Encounter rate (hares km^−1^)	Density (hares km^−2^)	95% CI	CV(%)
C (N)	23.3	119	96	4.1	25.6	19–35	15.9
C (D)	41.1	208	27	0.6	38.0	25–57	20.7
G (N)	15.3	46	106	6.8	41.8	31–57	15.2
G (D)	21.6	63	17	0.8	45.0	27–76	26.7

(N) – night time surveys.

(D) – daytime surveys.

For site C survey efficiency of daytime surveys was 0.33 that of night time surveys; for site G efficiency of daytime surveys was 0.29 that of night time surveys.

### Population density estimates

Hares were recorded at all seven sites but despite the fact that all sites were situated within the same region and were superficially similar in composition and management, densities varied widely between them (Table 2). The overall mean density was 20.6 hares km^−2^ (95% CI 18–23 km^−2^) with a post-breeding (autumn) density of 22.6 hares km^−2^ (95% CI 18–28 km^−2^) and a pre-breeding (spring) density of 18.9 hares km^−2^ (95% CI 15–23 km^−2^). Encounter rates varied between 0.4 and 11.1 hares km^−1^ (mean = 3.8; SD = 3.6; n = 7) for autumn and 0.5 and 6.7 hares km^−1^ (mean = 3.0; SD = 2.3; n = 7) for spring (Table 2). The minimum spring density estimate at any site was 2.9 hares km^−2^ (95% CI 1–7 km^−2^) while the highest was 39.9 hares km^−2^ (95% CI 25–64 km^−2^).

**Table 2:** Hare density estimates, total effort, observations and encounter rates in the autumn 2007–spring 2008 surveys in seven pastural sites in NE England (Fitted model was a Hazard-rate key function with a cosine adjustment term, with 16 m intervals, 130 m truncation).

Site	Total transects	Total effort (km)	Observations (hares seen)	Encounter rate (hares km^−1^)	Density (hares km^−2^)	95% CI	CV(%)
A	73	17.5	63	3.4	21.2	15–29	16.4
B	109	22.2	10	0.4	2.9	2–5	35.4
C	149	28.1	141	4.8	30.0	24–37	11.6
D	43	16.6	30	1.7	10.8	7–18	25.6
E	30	13.0	12	0.9	5.8	3–11	35.4
F	72	17.6	56	3.0	19.0	13–27	19.3
G	40	15.6	139	8.9	52.8	39–71	14.9
Pooled	516	130.6	451	3.3	20.6	18–23	7.81

### Behaviour

**Figure 3:**
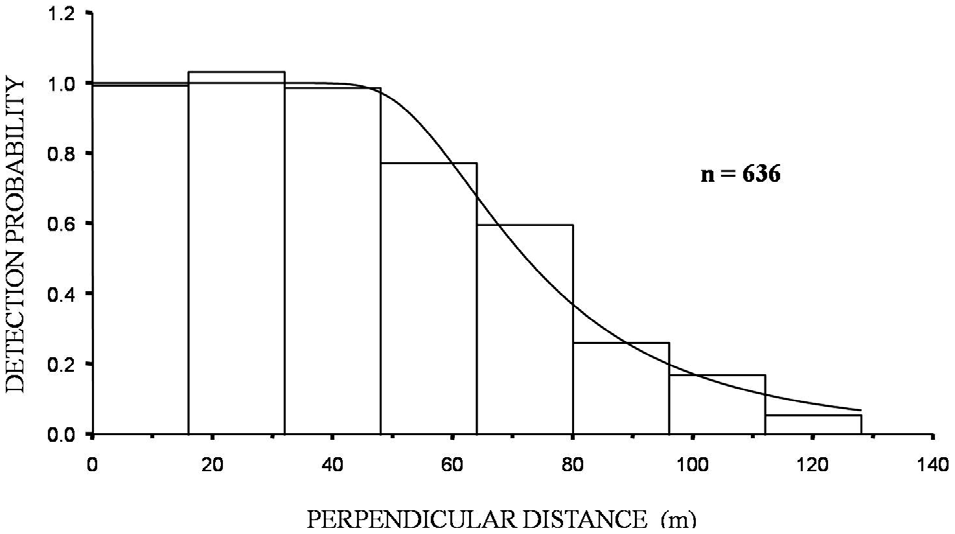
Histogram of total night time hare observations in all seven sites surveyed during 2007–2009. Fitted model is a Hazard-rate key function with a cosine adjustment term, with 16 m intervals, 130 m truncation.

**Figure 4:**
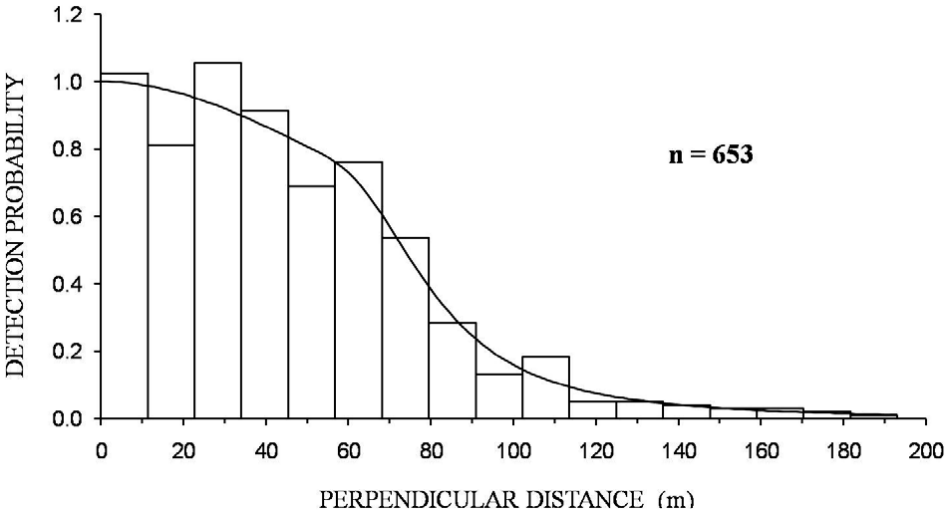
Histogram of total night time hare observations in all seven sites surveyed during 2007–2009. Fitted model is a Hazard-rate key function with a cosine adjustment term no intervals, no truncation.

Of all hares with recorded behaviour at the moment of detection (n = 577) only 10% were observed running while the majority (58%) were observed feeding, standing (15%), crouched (6%) or involved in both feeding and social interactions (10%). The small percentage of hares observed running suggests that most animals were detected at their original position, prior to evasive movement away from the observer, a fact also confirmed by the histograms of pooled night time observations (Figures 3 and 4).

In order to understand the effect of different daytime and night time encounter rates on hare detections at all sites we converted our observed (night time) encounter rates to hypothetical daytime encounter rates by dividing by the ratio between daytime and night time encounter rates from our comparison study in sites C and G (i.e. 7.65). We used these to compare the probability of detecting one or more animals on a 1 km transect were surveys to be carried out during the day or during the night. We used a simple Poisson model for hare encounters with the mean as the observed or hypothetical encounter rate for night time and daytime rates respectively (Table 3). While the Poisson model may not be suitable for hares at high densities, where encounters are likely to be clumped, we consider it to be a reasonable approximation for hares at these moderate densities and a useful didactic model in this case.

**Table 3:** Encounter rate and probability of encountering at least one hare on a 1-km transect at each site.

Site	Night time		Daytime	
	Encounter rate (hares km^−1^)	Probability of detecting ≥1 animal	Encounter rate (hares km^−1^)	Probability of detecting ≥1 animal
A	3.4	0.97	0.44	0.36
B	0.4	0.33	0.05	0.05
C	4.8	0.99	0.63	0.47
D	1.7	0.82	0.22	0.20
E	0.9	0.59	0.12	0.11
F	3.0	0.95	0.39	0.32
G	8.9	1.00	1.16	0.69
Overall	3.3	0.96	0.43	0.35

Daytime encounter rates were calculated by dividing night time encounter rates by the ratio between daytime and night time encounter rates at sites C and G (see Table 1).

Over all sites there was a high probability (96%) of detecting at least one hare within 1 km on night time transects, but a relatively low probability (35%) on daytime transects. Only the highest density site (site G, hare density 53.9 hares km^−2^) had a probability of detection >50% in daytime surveys, while all but the lowest density site (site B, hare density 2.9 hares km^−2^) had detection probabilities >50% in night time surveys.

### Detecting changes in population density

The decrease in number of hare observations between autumn 2007 and spring 2008 for all seven sites was reflected in a 16.4% decrease in the mean calculated density. This apparent decline in observations was most noticeable at the highest density site, G (Table 4). For most other sites density estimates were very similar between seasons and their overall autumn and spring densities were not significantly different (Z-test, Z = 1.158; P = 0.246). The overall detection function and the distribution of radial distances for all seven sites was marginally different for autumn and spring, with fewer observations near the transect line during spring, possibly as a reflection of increased activity during that time. At the highest density site, G, the detection function remained similar during both seasons. Extrapolating from the overall regional estimate of hare density of 20.6 hares km^−2^ (95% CI 18–23 km^−2^), derived from the pooled autumn and spring surveys, one further survey replicate might have been sufficient to detect population changes of around 25% (based on the Z test) if the precision of the new estimate were the same as the previous estimate.

**Table 4:** Estimates of hare density, effort and encounter rates for night time surveys at two sites during 2007–2009 (calculated with Uniform key function with cosine adjustment term, 16 m interval, 115 m truncation).

Site	Year	Season	Transects (number)	Total effort (km)	Observations (hares seen)	Encounter rate (hares km^−1^)	Density (hares km^−2^)	95% CI	CV(%)
C	2007	Autumn	78	13.9	77	5.5	32.9	24–45	15.9
C	2008	Spring	71	14.1	64	4.2	26.8	20–36	15.0
C	2008–2009	Winter	119	23.3	96	4.1	25.6	19–35	15.9
G	2007	Autumn	20	7.7	86	10.5	66.4	45–99	19.5
G	2008	Spring	20	7.9	53	6.5	39.9	25–64	23.0
G	2008–2009	Winter	47	15.3	106	6.8	41.8	31–57	15.2

For sites C and G, where between-year comparisons were possible, density estimates for winter 2008/2009 were almost identical to the ones obtained for spring 2008; for site C these were 26.8 hares km^−2^ and 25.6 hares km^−2^ respectively, and for site G 40 hares km^−2^ and 41.8 hares km^−2^ respectively. This was also reflected by the very similar encounter rates between spring 2008 and winter 2008 (Table 4).

## Discussion

Adequate population assessments can form a substantial basis on which to set conservation targets and repeated surveys are necessary to evaluate whether these targets can be or have been met. The methods employed in obtaining these assessments will be restricted by issues of suitability, logistical feasibility and cost, though there are many examples of monitoring programmes which operate inefficiently [20], [21]. Our results demonstrate how one aspect of a species, its detectability, can affect the results of surveys with potentially wide-reaching implications for setting and monitoring of conservation targets.

### Assessing hare populations

Nocturnal and crepuscular species, like brown hares, typically use different habitats throughout the 24-hour cycle. Brown hares use discrete patches for nocturnal feeding and daytime resting [22] and the latter can be located in areas unsuitable for distance sampling due to poor visibility or limited access, such as mature crops left standing over winter or dense woodland [15], [23]. Additionally, during daytime surveys the surveyor typically relies on flushing resting animals [14]. As is clear from our daytime survey data, this only happens when in close proximity to the surveyor, which results in a very narrow strip width being surveyed and not only a small number of observations (Tables 1 and 3), but also data from a very small proportion of the survey area with greater potential biases from non-random placement of survey transects. The robust nature of our estimates from night time surveys is supported by the very similar density estimates generated from a variety of models and narrow confidence limits from the best fitting model (Tables 2 & 4). A combination of low encounter rates and the narrow effective strip width in daytime surveys led to estimates with high variability. However, brown hares often feed in social groups during the night [24] and at the high end of hare densities in our sites (site G) night time line-transect surveys became less efficient due to the increased possibility of encountering aggregations of several individuals. Treating these as individual sightings violates the object independence assumption in Distance. Although the programme is considered very robust to such violations [25] it can result in increased variance due to large differences in encounter rates between transects and consequently poor precision, or difficult model fitting due to large ‘spikes’ in the distance histogram [26].

### Hare density estimates

Our average density estimate of 20.6 hares km^−2^ for the study region is several times greater than the average for brown hares from the national hare survey but is by no means extreme. Given the right conditions continental European populations of brown hares can reach densities in excess of 100 hares km^−2^ [11]. In Britain hare densities of 87.3 hares km^−2^ in ‘optimum conditions’ (the presence of predator control and habitat improvements in arable areas) and 28.5 hares km^−2^ in ‘suboptimal conditions’ (mixed rough pasture/arable areas with no habitat improvements and only 3 years of predator control) have been recorded [27]. Although in our study hare density was not clearly related to sites with predator control (sites B, C, D, G; [28]) it is likely that at least in some of the sites hare populations had benefited from measures put in place for game rearing, such as the presence of small blocks of woodland and herbaceous strips in field margins, as well as predator control, all of which have been shown to positively influence hare densities [27], [29]. While it is possible that hare numbers in our study region have increased since the time of the national survey, we propose an alternative explanation for the discrepancy and which is relevant to widespread studies of any species with low daytime detectability: that daytime surveys result in under-sampling and consequently, across wide spatial scales, underestimating density. The results from our seven sites show variability in population density greater than a factor of ten between superficially similar nearby sites, all dominated by the same pastural habitat, (e.g. sites B, C and G, Table 2). The low encounter rate during daytime surveys means that the probability of detecting hares in these surveys, even at relatively high densities, is remarkably low over 1 km transect walked. At site A, where estimated densities were approximately equal to the British average for pasture land, detection probability in a 1-km transect was 5% in the daytime, and at site C, with density over 10 times higher, detection probability was 46%. Extrapolating these figures to the national hare survey, where participants surveyed 3 km in each site on 3 occasions in each of the two years, the estimated probability of detecting one or more hares at our site B (2.9 hares km^−2^, just below the national average for pastures) in any one daytime survey would be 19% and over the course of all surveys and both years would be 59% (assuming a binomial distribution of detection probabilities and independence between temporal replicates at a site).

Mean daytime estimated density was higher than night time although with poorer precision. However, the greatly reduced encounter rates during daytime surveys imply that in many sites where hares are present at modest or low densities, even extensive daytime surveys risk not detecting their presence. Regional or national hare density estimates based on such data would rely on a weighted average of those sites where hares were encountered and those with null counts, artificially reducing the overall estimate. Furthermore, distribution maps would likely indicate low levels of presence at regional scales where in fact hares might be widely, if sparsely, distributed. This problem would be exacerbated by a low-efficiency sampling method requiring individual sites to be surveyed multiple times in order to generate sufficient encounters.

### Implications for future monitoring programmes

The national hare survey identified broad scale patterns in the distribution and abundance of hares, but was unable to resolve hare densities at scales that were useful to local or regional land and conservation managers. It has therefore been impossible to evaluate whether the management interventions that were intended to increase hare numbers, such as conservation headlands and set-aside [8], [22], that can benefit hares at farm level [30] have had any impact at regional or national levels (http://www.ukbap-reporting.org.uk/). Our analysis indicates that a straightforward modification of the methods of the national hare survey is far more likely to provide appropriately detailed data to overcome this problem. There has been no recent repeat of the two national hare surveys, perhaps because of the significant effort and cost involved in co-ordinating its 550 volunteers [13] but brown hare numbers are being recorded as part of a number of wildlife monitoring schemes [6]. While such schemes might provide information on general trends, particularly for culled populations, the fact that hares were only recorded in 2.6% to 30.5% of all squares surveyed in these schemes [6] suggests that the same issues of low detection probability will restrict the value of these schemes at regional or national scales.

## Methods

### Study site

Line transect distance sampling was carried out at seven study sites (named A–G) located in a lowland/marginal upland (30–250 m above sea level) pastural landscape in North Yorkshire, north east England. A detailed description of the sites is given in Petrovan *et al.* [28]. All sites were dominated by extensively grazed sheep *Ovis aries* and cattle *Bos taurus* pastures with some fields used primarily for silage production. Six of the seven sites were surrounded by more pastures and/or moorland; one, site G, was surrounded by a mixed arable-pastural landscape. Some form of shooting (mainly of pheasants *Phasanius colchicus*) took place at most sites but only sites B, C, D and G included a permanent gamekeeper, responsible for enforcing predator control in order to protect the stock of birds for shooting. However, even in these sites there were farms where no form of hunting or predator control was permitted. Hares were actively hunted with relatively low intensity in sites C, F and G.

### Sampling design

Sites were selected through inspection of remotely sensed images in Google Earth (http://earth.google.com) based on the identification of large grassland areas supported by ground truthing. No information on hare densities in any of the sites was available prior to the start of the surveys and site selection was therefore entirely independent from hare density. Field transects were established along ‘transect routes’ composed of consecutive fields; each route incorporated between 6 and 22 fields (mean 9). Sites had between two and six transect routes, each 2–3 km long. Transects comprised the entire length of each field on the transect route and were surveyed by walking a straight line through the middle of the field. This aimed to minimize the effect of field boundaries, such as tall hedgerows or walls, that otherwise create ‘no visibility’ habitat subzones thereby introducing significant ambiguity and resulting in potential density underestimations of 10–70% [31]. Routes were designed to cover as much of each site as possible given the constraints of accessibility and access permission. Less than 5% of >60 landowners refused permission, hence coverage was reasonably comprehensive and representative of the landscape with no obvious geographical or landuse bias. In order to ensure independence of detections transect routes were a minimum of 300 m apart or separated by substantial natural or artificial barriers such as streams or robust fences. Routes did not deliberately follow any landscape or manmade features, such as streams, valleys, roads or foot paths.

### Data collection

#### Night time versus daytime surveys

Two sites (C & G) were selected for an evaluation of the differences between day and night time surveys. Transects were walked twice during night time and three times during daytime using the same network of transects. These surveys were carried out during autumn and winter seasons (October to March 2008–2009) but for simplicity here will be referred to as ‘winter’ surveys.

#### Population density estimates

From autumn 2007 to spring 2008 we surveyed all transects at all seven sites twice during the night; once between October and December 2007 (hereafter ‘autumn surveys’) corresponding with the end of the hare breeding season when population levels should be at their maximum [13] and the second time between January and March 2008 (hereafter ‘spring surveys’), after peak winter mortality when the adult population should be at a minimum. However, due to the difficult weather conditions, with extensive rain and fog in spring 2008, surveys had to be extended into April and early May for a small number of transect routes. In rare instances some fields could not be included in all surveys due to unexpected events, such as the presence of adult bulls in the field.

All night time surveys were started at least one hour after sunset and finished before 23:30 hours, while daytime transects were only walked between 10:00 and 14:00 hours when most hares would be inactive in their forms [32], [22]. Days with poor visibility due to fog or heavy rain and particularly cold nights or those with bright moonlight were avoided as these negatively influence the proportion of active hares at night [33], [34]. Surveys were carried out by two people walking slowly and silently along the transect scanning an arc of 180° with a 1 mega-candlepower spotlight (Clubman CB2, Cluson Engineering Ltd, Hampshire, UK) and 8×42 binoculars. Observations of animals were made by the same observer to avoid between observer differences. A trained and experienced observer collected all daytime data and these were independently verified (by S.O. Petrovan who collected all night time observations) on 15% of all transects to ensure repeatability and consistency of results. All sites were surveyed on foot to avoid contravening the recommendation that transects do not follow existing linear structures such as roads or paths [17], [26] and to be able to include fields irrespective of road access.

In order to assess whether hares were reacting to the observer prior to detection, the behaviour of each individual sighted was recorded.

During night time surveys all hares that were displaced by the surveyors were followed with the lamp as they moved away to establish with precision the direction of movement in order to minimise the probability that animals would be counted twice while moving through successive fields. Hares were subsequently ignored if they relocated to other fields that were on the transect route and as such were seen for a second time. Transect sections were recorded using GPS and superimposed on 1∶10,000 Ordnance Survey maps in ArcGIS version 9.1 (ESRI California, USA) in order to calculate total distance walked. Distances to sighted animals were measured using a laser range finder (Leica LRF900, Germany) with 1 m precision and 7× magnification. Angles between the transect line and the location of the observed animal were measured using a compass to the nearest degree. Typically hares were easily distinguished in the field from rabbits, which were largely sympatric, but in cases when identification was problematic angle and distance were recorded and subsequently the animal was approached by the observer until the species could be identified with certainty.

### Data analysis

A detailed explanation of the theory of distance sampling and practical aspects of the analysis is provided by Buckland *et al.* [26]. Program DISTANCE version 6.0, release 2 [[25], http://www.ruwpa.st–and.ac.uk/distance] was used to calculate the density and abundance of brown hares in the surveyed sites. Detection functions were calculated across sites for each season and pooled seasons while specific density estimates were generated by post-stratification at the levels of site, season or year. Where counts were sufficient (i.e. >40 observations) a separate detection function was calculated for each site to investigate the differences in the detection of animals in different areas and at different densities. Since all surveys took place in the same habitat type (grassland) and with the same species, for comparisons between daytime and night time surveys separate detection functions were generated for each of these pooled across sites and seasons. Global density was calculated as the mean of stratum estimates weighted by stratum area [26], [25]. No left truncation or the forcing of data into arbitrary bins was applied but instead a combination of analysis using ungrouped data or data grouped at equal intervals and different right-hand truncations (0%, 3%, 5%) were used in order to select for the model with the best fit. Distance data were modelled by fitting three key functions and three series expansions to the data [26]. Model performance was evaluated using a combination of Quantile quantile (q-q) plots, Goodness of fit tests, Kolmogorov-Smirnov and Cramer-von Mises family tests for ungrouped data and Akaike's Information Criterion (AIC) for both grouped and ungrouped data as well as truncated or not truncated data, with the best model, in terms of parsimony, selected on the basis of the lowest AIC value. Variance was calculated using a combination of the default empirical calculation and in some instances an advanced analytic encounter rate option with post-stratification of overlapping strata made of adjacent samples. This method was shown to give more robust estimates of variance when there were strong spatial trends in the studied area [35]. In this case consecutive field transects rather than parallel systematic lines were used in order to account for the variance in encounter rate. Comparisons of independently obtained density estimates between seasons, years or between night time and daytime surveys were performed using the *z test* [26].

We assessed the relative efficiency of daytime versus night time surveys by comparing coefficients of variation using the formula:
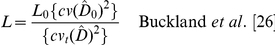
where L is the total transect length, L_0_ is the transect length covered in the study, *cv*(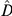) is the coefficient of variation in the pilot study and *cv_t_*(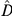) is the target value for the coefficient of variation. The relationship between survey efforts required for two sets of surveys to achieve equivalent coefficients of variation (i.e. *cv_tN_*(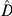) = *cv_tD_*(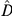)) can be described by:
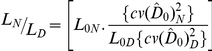
This we define as the ‘survey efficiency ratio’.
